# Review of Associated Health Benefits of Algal Supplementation in Cattle with Reference to Bovine Respiratory Disease Complex in Feedlot Systems

**DOI:** 10.3390/ani12151943

**Published:** 2022-07-30

**Authors:** Marnie Willett, Michael Campbell, Ebony Schoenfeld, Esther Callcott

**Affiliations:** 1 School of Animal, Environmental and Veterinary Sciences, Charles Sturt University, Wagga Wagga, NSW 2650, Australia; mjwillett@bigpond.com (M.W.); corriedoun@gmail.com (M.C.); eschoenfeld@csu.edu.au (E.S.); 2 Graham Centre for Agricultural Innovation, Charles Sturt University, Wagga Wagga, NSW 2650, Australia

**Keywords:** algae, fatty acids, oxidative stress, immune-modulation, respiratory disease, bovine, *Nannochloropsis oculata*

## Abstract

**Simple Summary:**

Bovine Respiratory Disease is considered one of the most common diseases within the Australian beef industry. Research into the use of algal supplementation is increasing due to the positive health benefits associated with the bioactive compounds within the algae. Studies have found benefits such improving the animal’s immune response to the disease and overall health. Further research is required to fully investigate these results.

**Abstract:**

Within the Australian beef industry bovine respiratory disease is considered one of the most common disease and costs the industry an average net loss of $1647.53 Australian dollars per animal death to bovine respiratory disease complex (BRD). This is due to the disease overwhelming the animal’s immune system during a period where they experience multiple stressors that consequently increase the animal’s susceptivity to disease. Research into the bioactive compounds commonly found in marine algae is rapidly increasing due to its positive health benefits and potential immune modulating properties. Algal supplementation within previous studies has resulted in improved reproduction potential, growth performance, increases antioxidant activity and decreased proinflammatory cytokine concentrations. Additional research is required to further understand the aetiology of BRD and complete analysis of the bioavailability of these bioactive compounds within marine algae to fully explore the potential of marine algae supplementation.

## 1. Introduction

The Australian beef industry is a major contributor to the economy, representing approximately 20% of the total livestock production and producing over 2% of the worlds beef supply [1]. There are approximately four-hundred registered feedlots within Australia which intensively feed a high-energy concentrate diet to cattle aged between 16 and 20 months of age [2]. These feedlot systems are designed to increase the body weight of the animals in order to meet various market requirements for either global exportation or Australian distribution [3]. During the transitioning period of young cattle going into the feedlot system they experience multiple stressors such as environmental changes, dehydration, and fatigue. These stressors negatively impact on the animals’ overall health by markedly increasing physiological stress and decreasing immune response making them more susceptible to diseases, such as bovine respiratory disease complex (BRD) [2,4].

Furthermore, known as shipping fever, BRD, is a respiratory disease that affects the upper and lower respiratory tracts of cattle. It is the most common cause of clinical disease and death in Australian beef feedlots [2,5,6]. BRD aetiology is multifactorial that is instigated by a combination of factors including physiological stress and disease-causing agents such as bacteria. It has been noted in the literature that single factors alone, do not contribute to the pathogenesis of BRD [2]. It can affect cattle at any age and stage of production. Chamorro and Palomares [7] identified cattle populations commonly affected by BRD were dairy calves less than three months of age, pre-weaning beef calves younger than five months of age, and weaning beef calves between five and eight months of age.

Within the Australian feedlot industry, BRD has been found to negatively impact on animal production and profitability. Blakebrough-Hall et al. [8] observed that BRD caused significant financial losses due to decreased production and increased treatment costs. Furthermore, the disease has an 18% morbidity rate and a 2.1% mortality rate, with an average net loss of $1647.53 AUD per BRD mortality. Overall, BRD has been estimated to be the cause of 70% of clinical disease cases and 50% of deaths in Australian beef feedlots [9]. In conjunction with this, animals that recovered from BRD returned, on average, between $67.10 and $213.90 AUD less at slaughter compared to healthy animals due to a lighter carcass weight [8]. With current industry practice relying solely on preventative measures such as vaccination and medical treatment of the disease, there is an increased interest in the use of nutraceuticals to compliment current preventative measures and increase immune responses within the feedlot industry [10,11].

Due to the presence of bioactive compounds, research into the use of algae has been increasing within the poultry [12], fish [13], pig [14] and cattle industries [15,16]. There is special interest in these biologically active compounds which include omega fatty acids, polyphenols, polysaccharides, and chlorophyll, due to their positive health benefits [16,17]. In beef cattle, algae supplementation has been demonstrated to have multiple benefits such as improved weight gain, increased growth rates [18], improved immune responses [10], as well as having antibacterial, antiviral properties and antioxidant properties [16,19].

*Nannochloropsis oculate* is a green algae variety rich in long chain polyunsaturated fatty acids (LC-PUFA) including omega-3 and omega-6 fatty acids [20]. It has been shown to contain the omega-3 fatty acids docosahexaenoic (DHA, C22:6 n-3) acid and eicosapentaenoic (EPA, C20:5 n-3) acid [21,22]. These fatty acids can have powerful anti-inflammatory and immunomodulatory activities in a wide array of diseases [23]. To assess the potential of *N. oculata* as a dietary supplement to reduce the risk of BRD within the feedlot requires additional knowledge. Research into the aetiology of BRD, how enhancement of the immune system can decrease the risk of BRD as well as the immunomodulatory effects of *N. oculata,* are all key factors that should be further explored.

## 2. Bovine Respiratory Disease

**Figure 1:**
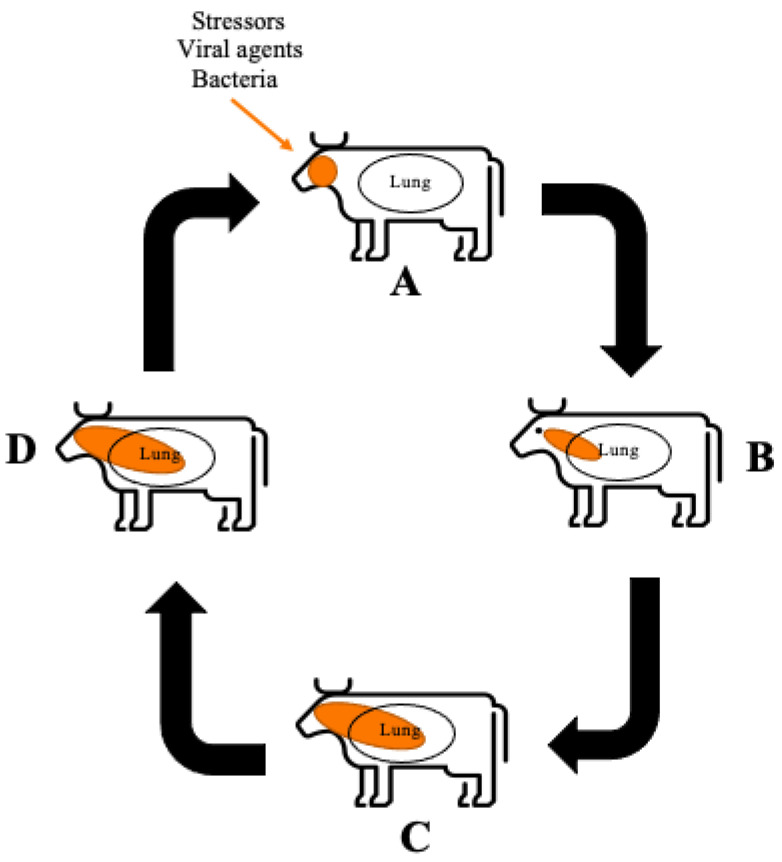
**Schematic overview of BRD progression** (**A**) The immune system is challenged allowing bacterial and viral infection to occur. Function of the ciliated epithelial cells is inhibited by necrosis or dysfunction. The immune system is activated via the PRR recognition of PAMPs. Neutrophils are recruited to the area as the first line of defence. (**B**) BRD causing bacteria proliferate in the upper respiratory area allowing infection of the bacteria into the lower respiratory area. The PRR recognise the PAMPs produced by the pathogens. Respiratory epithelial cells which contain TLR and NOD-like receptors also recognise PAMPs and in response produce pro-inflammatory cytokines. (**C**) Pro-inflammatory cytokines recruit a larger neutrophil response. Neutrophils undergo NETosis producing NETs stimulating cell death of surrounding pulmonary epithelial cells, resulting in pulmonary damage. (**D**) Persistent infection of BRD can occur through *M. haemolytica* producing a leukotoxin that binds to the [beta]_2_ integrin impairing the function of leukocytes, resulting in apoptosis, allowing the bacteria to evade host detection.

The aetiology of BRD has still not been fully elucidated [24]. Current literature suggests a variety of factors, including stress, viral and/or bacterial agents, can synergistically contribute to overwhelm and dysregulate the animal’s immune system resulting in disease [2,25]. Figure 1 depicts a schematic overview of how BRD progresses within healthy cattle from initial infection; to the resulting pulmonary damage reported in BRD affected animals and the persistent infection that can occur in adult cattle. Figure 1 demonstrates that factors such stressors, viral agents and bacteria enter the animal and instigate necrosis or inhibition of the ciliated epithelial cells resulting in inability to remove pathogens from the upper respiratory tract. Consequently, BRD causing bacteria colonise and proliferate in the upper respiratory tract and into the lower respiratory tract. An immune response is triggered by the pattern recognition receptors (PRR) recognising the pathogen-associated molecular patterns (PAMP) which are produced by the BRD causing bacteria, this response includes the recruitment of neutrophils to the area as the first line of defence [25]. In conjunction, respiratory epithelial cells containing Toll-like receptors (TLR) and nucleotide oligomerisation domain (NOD)-like receptors are activated in the presence of PAMP, resulting in the production of pro-inflammatory cytokines [26]. The large production of pro-inflammatory cytokines and subsequent recruitment of neutrophils leads to the production of neutrophil extracellular traps (NET) which are capable of neutrophil-mediated pulmonary tissue damage by stimulating cell death of pulmonary epithelial cells [25].

Ferraro et al. [27] identified that there was no clear definition of clinical symptoms associated with BRD, which made it difficult to diagnose from the signs exhibited by animals. Signs such as depressed mentation are often masked in cattle, due to their evolved survival mechanisms as prey animals, making the identification BRD difficult, resulting in low diagnosis rates [28]. Ferraro et al., (2021), later defined the clinical signs of BRD to include dyspnoea, tachypnoea, pyrexia, in addition to nasal discharge and coughing.

A consequence of BRD is pulmonary damage, due to inflammatory mediators contributing to the development of oedema and fibrous polyps within the lungs [29]. Angen et al. [30] showed that in 68% of the bronchoalveolar lavage fluid samples, collected from clinically healthy calves, tested positive for bacterium which are associated with the causation of BRD. With the natural presence of these bacteria in healthy cattle, any physiological stress to the animal has the potential to lower immune responses allowing the bacteria to overburden the animal. As a result, bacterial invasion has the potential to facilitate the pathogenesis of BRD due to alterations of the respiratory mucosa and immune system [26].

Stressors linked to BRD such as extreme temperatures, dust, injury, fatigue and dehydration are generally experienced when animals are transported to the feedlots, and also whilst at the feedlot [25]. Taylor et al. [31] reported sorting, loading and early transit were likely the most stressful components due to fasting, handling and potential injury resulting from handling and/or loading onto transportation. Human error has also been found to contribute to increased clinical implications such as dehydration in cattle due to lack of access to water when experiencing delayed transit or prolonged transportation. This is due to fatigued workers causing delays in processing the cattle into the feedlots and hence extending exposure to environmental stressors [31].

Viral agents predispose cattle to BRD in two distinct ways [25]. Firstly, the viral agent itself causes direct damage to respiratory clearance mechanisms by inhibiting function or activity of ciliated respiratory epithelial cells. Ciliated respiratory epithelial cells are responsible for trapping and expelling bacteria via the throat; preventing the bacteria from the deeper lung regions which remain relatively sterile in healthy cattle [26]. Viral agents inhibiting immunoprotective functions enables bacteria to proliferate to levels capable of causing disease [26,31]. Secondly, viral infections can interfere with the ability of the immune system to respond to bacterial infection. The viral infection interferes with the immune system through induction of apoptosis of specific T helper cells and their cell surface molecule CD4 [32].

Bacterial agents such as those listed in Table 1, have the capability to cause BRD through colonisation within the tonsil and mucous of the nasal passages due to ease of aerosol transmission during transport and in pens with animals in close proximity of others [26]. *M. haemolytica* is the major pathogen significantly associated with the pathogenesis of BRD, due to its high prevalence rate in clinically diagnosed cattle. It is suggested this is due the bacterium’s increasing antimicrobial resistance and the desirable conditions created when the animal is exposed to stressors allowing it to proliferate in the lung [33].

### Airway and Lung Epithelia

The respiratory tract of cattle differs to other animals due to its relatively long tracheobronchial tree and associated increased dead space within the lung [26]. This dead space means that the gas transit time within the lung and the surface area is increased allowing for particulate matter to deposit creating opportunity for bacteria to proliferate in ideal growing conditions of a relatively warm and moist area.

The respiratory tract is considered the first line of defence of the animals’ innate immune system as it is where the bacteria initially come into contact with the animal. Bacteria are inhaled as droplets via aerosol transmission into the nasal passages where they can adhere and colonise the epithelial surface [6]. The epithelial cilia of the trachea are responsible for the removal of pathogens; however, viral agents such as BRSV and PIV can cause ciliary dysfunction and necrosis delaying the removal of bacteria from the airway [25]. Upper respiratory tract damage is also caused by BHV-1 by infecting the epithelium layer subsequently causing rhinotracheitis. The lesions caused effectively destroy the normal processes of pathogen removal via inhibiting cilia movement and, allowing commensal bacteria such as *M. haemolytica* to colonise and proliferate in the lower respiratory tract causing disease [32]. Furthermore, dehydration has also been shown to disrupt the air-surface liquid lining (ASL) within the upper respiratory tract by increasing the viscosity of the lining. As a result, this decreases the animal’s ability to trap pathogens entering the respiratory system and to eliminate particulates via coughing or sneezing [26].

## 3. Immune Responses

The immune system is responsible for recognising the PAMPs produced by the BRD causing pathogens. The response involves the production of pro-inflammatory cytokines from infected pulmonary epithelial cells in conjunction with neutrophils to the site of infection to mount the appropriate antibody response. It is also this immune response that contributes to the pulmonary tissue damage reported in infected cattle [5,26]. However, BRD causing bacteria *M. haemolytica* is able to evade host detection by producing a cytolytic exotoxin resulting in persistent infection within adult cattle [32].

### 3.1. Pro-Inflammatory Cytokines

With the immune system activated, neutrophils, and monocytes are recruited to the site of infection in response to pro-inflammatory cytokines [25], such as interleukin-1 (IL-1), interleukin-8 (IL-8), and tumour necrosis factor-α (TNFα) [5,26]. With the recruitment of these cytokines there is also the attraction of dendritic cells, NK, T, and B cells as part of the antibody response to the infection [25]. In particular, the chemokine receptor CXCR1 gene as identified by Lindholm-Perry et al. [34], contributes to the attraction and activation of neutrophils at the site of inflammation when stimulated by IL-8. Pro-inflammatory cytokines, whilst being the main promoters for effector element development within the immune response [32], also have an important role in the physiological signs exhibited by the animal as they are capable of crossing the blood–brain barrier allowing the modification to the animals thermoregulation causing fever, as well as the ability to modify behaviour creating a desire to sleep [35]. These are the typical clinical signs as observed by cattle with BRD. It has been suggested that the pulmonary damage seen in BRD affected cattle is due to the rapid secretion of pro-inflammatory cytokines triggering a large recruitment of neutrophils to the site of infection. Coinfection of BRD causing bacteria with viral pathogens has also been shown to intensify this cytokine production [25].

M. haemolytica produces an exotoxin that is cytolytic to white blood cells and has hence been termed as a leukotoxin [32]. During the bacteria’s growth phase, leukotoxin production is at its peak, causing leukocytes to release eicosanoids and cytokines. Prolonged periods of high leukotoxin levels impair the function of the leukocytes resulting in apoptosis [36]. The leukotoxin is able to cause the destruction of leukocytes due to its ability to bind to the leukocyte specific integrin [beta]_2_. The [beta]_2_ integrin is a transmembrane protein that allows the leukocyte to perform functions of phagocytosis, antigen presentation and focusing in on areas of inflammation [32]. M. haemolytica binding properties facilitates evading host detection and reduces the production of pro-inflammatory cytokines and may partially explain the persistent infection seen in adult cattle [37].

Ozkanlar, Aktas, Kaynar, Ozkanlar, Kirecci and Yildiz [37] describes cytokine levels as significantly elevated after initial infection with a BRD causing bacterial agent with levels decreasing once the calves began exhibiting clinical signs such as fever and tachypnoea. This is also supported by studies performed by Burciaga-Robles et al. [38] reporting an increase in cytokine levels within cattle exposed to M. haemolytica and also recorded an increase in Interleukin-1β (IL-1β) in persistently infected cattle. Both authors recognise that exposure to BRD causing agents result in an increase in pro-inflammatory cytokines which contributes to extensive pulmonary tissue damage. However, further investigation is required to evaluate the long-term effects of BRD in cattle.

### 3.2. Neutrophils

The neutrophil is one of the first cell types to be recruited to the site of infection due to its phagocytic role of consuming foreign substances. Whilst they have an important role in the removal of extracellular bacterial infections, neutrophils are also considered a contributor to the pulmonary tissue damage caused by BRD infection as they contribute to the formation of intralobular oedema, alveolar necrosis and occlusion of the small airway [25,39,40]. This damage is due to neutrophils releasing NETs, which contain extracellular deoxyribonucleic acid (DNA) and proteins that stimulate a specific cell death process known as NETosis [25,41]. NETs have been reported to contribute to neutrophil-mediated pulmonary tissue damage by stimulating cell death of pulmonary epithelial cells. Breider, Walker, Hopkins, Schultz and Bowersock [39] reported calves with induced neutropenia, using hydroxyurea, had a markedly decreased number of alveolar lesions compared to calves with no neutrophil alterations. Radi et al. [42] supports these results with a similar study, demonstrating a decrease in pulmonary lesions within calves that were subjected to induced neutropenia. This supports the concept that neutrophils do play an active role in the pathogenesis of BRD by exacerbating pulmonary tissue damage in the presence of pathogens.

### 3.3. Pattern Recognition Receptors

Located within the innate immune system are PRRs which recognise PAMPs [25]. All pathogens within the BRD complex produce some type of PAMP that activates the innate immune system within the animal. In acute inflammation, neutrophils also recognise PAMPs, which stimulates other immune cells including natural killer (NK) T cells, T cells, B cells, and plasmacytoid dendritic cells to interact with the PAMP through activation of PRR. Furthermore, respiratory epithelial cells containing Toll-like receptors (TLR) and nucleotide oligomerisation domain (NOD)-like receptors are activated in the presence of PAMP, resulting in the production of pro-inflammatory cytokines [26]. The immune systems recognition of PAMPs within the body allows for the necessary immune response to BRD. However, the recruitment of pro-inflammatory cytokines and neutrophils as a result of PAMP recognition within the immune system is also a contributing factor to the large immune response to BRD bacteria that is capable of causing pulmonary tissue damage as previously described.

## 4. Bioactive Compounds Found in Algae

**Figure 2:**
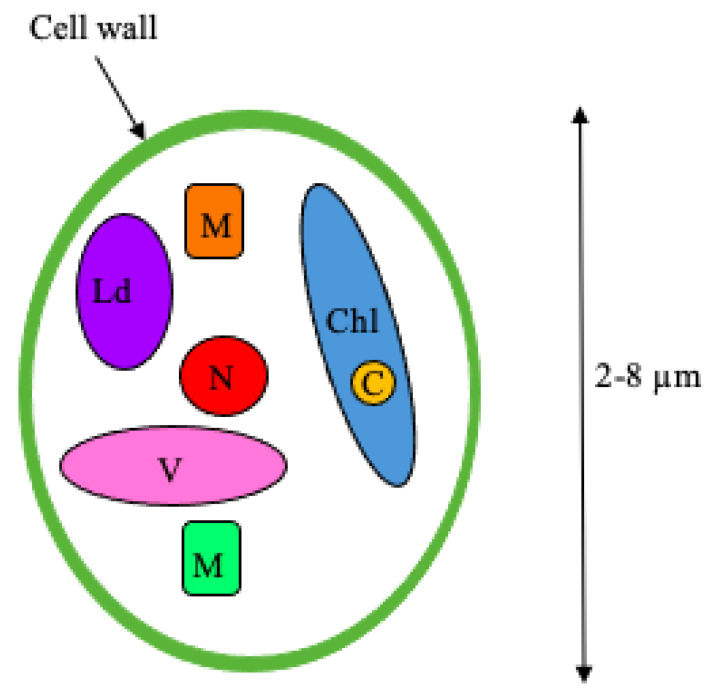
**Basic structure of *N. oculata*.** This graphical representation depicts the basic structure of *N. oculata*. It is comprised of a fibrous skeleton containing a single chloroplast. The cell wall contains polysaccharide, protein, and lipid components packed in a shapeless mucilaginous material. M: mitochondria; Ld: lipid droplet; N: nucleus; V: vacuoles; Chl: chloroplast; C: chlorophyll.

Marine algae is becoming widely studied for its health benefits due to its bioactive compounds. Marine algae has been shown to contain proteins, polysaccharides, lipids, antioxidants and sterols [16,43]. Found in both marine and freshwater, *N. oculata* is a unicellular green alga with a polysaccharide cell wall structure, which comprises of a fibrous skeleton, containing a single chloroplast [44,45,46]. The cell wall consists of the polysaccharide, protein, and lipid components packed in a shapeless mucilaginous material [47]. The lipid droplet is also an important structural component as it provides energy to the organism itself [48]. The full structure of *N. oculata* is not well documented within the literature with specific cell wall structures still unknown [49]. Figure 2 shows the simple structure of *N. oculata* to depict the general arrangement of its components such as the single chlorophyll. The concentrations of the bioactive compounds within the alga vary with the environmental conditions the alga is grown in. This includes environmental factors of the water such as temperature, nutrients available within the water, light intensity [17], nitrogen availability, and salinity levels [50].

### 4.1. Fatty Acids

Fatty acids (FA) are the most abundant group of lipids to occur within algae which have multiple health benefits due to their anti-inflammatory and anti-bacterial properties [16]. The Nannochloropsis spp. are reported by Figueiredo, da Costa, Silva, Domingues and Domingues [21] to contain 19.15% of polyunsaturated fatty acids (PUFA) in dry biomass, with EPA concentrations as high as 12% dry weight. Adarme-Vega et al. [51] report EPA accounts for 39% of total FA within Nannochloropsis spp. Although DHA and EPA are the main fatty acids within algae, arachidonic acid (ARA), alpha-linoleic acid (ALA), linoleic acid (LA), and gamma-linolenic acid (GLA) also contribute to the total FA content [17].

### 4.2. Polysaccahrides

Described by Chojnacka, Wieczorek, Schroeder and Michalak [16] as one of the most important components of algae, polysaccharides have a wide range of health benefits including regulation of immune responses. The content of polysaccharides found within algae is dependent on the type; however, Holdt and Kraan [52] estimate the total polysaccharide concentration in seaweed species can range from 4 to 76% of dry weight. Chojnacka, Wieczorek, Schroeder and Michalak [16] similarly report the highest concentration of 76%. Pandeirada et al. [53] identifies that the polysaccharide content within marine algae consists of 19% (*w*/*w*) of the total biomass tested. Within this mannitol and glucans were the highest recordings of 34 and 36 mol%, respectively.

### 4.3. Polyphenols

Due to their antioxidant and radical-scavenging activity polyphenols are capable of both inactivating reactive oxygen species (ROS) and preventing the generation of free radicals which both have the potential to cause cellular damage when large quantities are allowed to form within the body [16]. Whilst all species of algae contains some content of polyphenols, the composition ranges anywhere from 1% to 10% dry matter. Green algae is suspected to have fewer phenolic compounds when compared to red or brown algae [16,54]. Ebrahimzadeh et al. [55] report *N. oculata* having a total phenolic content of 30.94 ± 1.61 mg methanolic extract. Metsoviti et al. [56] reported that growth conditions of high light intensity and temperature increased the phenolic compound content with the highest results of 2906 μg gallic acid equivalent (GAE) g^−1^ of dry biomass.

### 4.4. Chlorophyll

Chlorophyll is the green lipid-soluble pigment that enables plants and algae to perform photosynthesis [52]. *N. oculata* contains only one pigment of chlorophyll, chlorophyll a, which can reach levels as high as 6% dry weight as reported by Lubian, Montero, Moreno-Garrido, Huertas, Sobrino, Gonza’lez-del Valle and Pare’s [50].

## 5. Bioavailability of Algae Fatty Acids in Cattle

Bioavailability refers to the quantity of a substance to become physically attainable from the time of ingestion to the degree of systemic uptake [57]. The rate of gastrointestinal absorption of algae supplements in cattle is affected by the digestion process as this is a multi-step process involving the mechanical, biochemical and microbial processes that occur within the digestive tract of the animal [58].

Ruminants have a complex digestion system that involves many different processes that can affect the absorption and bioavailability of compounds, especially that of fatty acids due to extensive biohydrogenation by rumen microbes [59]. Glasser et al. [60] report that due to extensive biohydrogenation the unsaturated fatty acids within the rumen are not an accurate representation of the fatty acids that are available for the animal. Dietary lipids initially undergo lipolysis to release non-esterified fatty acids (NEFA), which subsequently undergo biohydrogenation to become monosaturated fatty acids (MFA) and saturated fatty acids (SFA) [61]. This leads to the loss of most PUFA within the algae supplement, such as omega-3, and the overall efficiency of the PUFA transfer from the diet to animal’s product (e.g., meat or milk) [62]. The NEFA produced are mainly metabolised in the liver where they are either esterified and secreted as very low density lipoproteins, esterified and stored intracellularly, oxidised completely to carbon dioxide, or oxidised partially to acetone or ketone bodies [63].

There are limited studies on the bioavailability of omega fatty acids in cattle, with studies focusing mainly on their content within animal products. Alves et al. [64] reported that n-3 long chain PUFA content in meat products of ruminants was relatively low at (2–40 mg/100 g) without supplementation of *N. oculata*. Similar levels are also reported by Flakemore et al. [65] with dietary supplementation of degummed crude canola oil. Increasing concentrations of fatty acids with results showing an increase from 17.9 mg/100 g to 21 mg/100 g within the muscle of sheep. Interestingly, these studies did not report on the availability of fatty acids within the plasma post supplementation. Further bioavailability studies are required with algae supplementation to evaluate the concentrations of fatty acids in plasma and muscle after the rumen biohydrogenation process to effectively correlate the rate of absorption from the digestion system.

## 6. Health Properties of Algae-Derived Bioactive Compounds

Algae supplementation in cattle is being widely considered as a nutraceutical alternative to fish oil [66] to compliment current BRD preventative measures such as vaccinations [24]. The use of algae may also assist with the industry demand of having a high quality and low-cost feed solution [67]. Within the Australian beef industry, studies have focused on how different algae species are improving outcomes such as daily weight gain, feed efficiency and animal product quality [68,69].

### 6.1. Increased Reproduction Potential

The provision of fatty acids through algae supplementation is increasing in popularity and generating research interest within the cattle industry. This is due to its benefits positively impacting on reproduction through enhancement of hormone profiles, ovarian function, oocyte quality and embryo development [70]. Diets containing omega-3 FA are reported to improve reproductive efficiency in dairy cows by inhibiting uterine secretion of prostaglandin F_2α_ (PG F_2α_). This is due to antagonism with arachidonic acid, which is a precursor for prostaglandin [22,66]. It is this inhibition of PG F_2α_ that has been speculated to enhance embryo survival at the time of pregnancy recognition when, achieved through dietary supplementation [22].

Supplementation of DHA to dairy cows have also revealed some positive results. Sinedino et al. [71] showed an increased proportion of oestrous cyclicity and pregnancy at the first artificial insemination (AI) by 39% when compared to the control group who did not receive any supplementation. A 22% increase in pregnancy rates was recorded in multiparous cows, which was almost doubled in primiparous cows. This is due to the DHA-rich algae increasing the fatty acid composition in plasma phospholipids aiding in the establishment and maintenance of pregnancy in dairy cattle. These results indicate algae supplementation has the potential to increase reproductive rates and efficiency of breeding cattle.

### 6.2. Increased Growth Performance

Beef enterprises have identified the need for a forage that is high quality, relatively low cost, readily available and capable of meeting market specifications of younger, heavier cattle at slaughter [67]. *N. oculata* can grow in a variety of environments and has very high growth rates, capable of doubling its biomass daily [72]. Therefore, may be a suitable supplement to add to cattle’s basal diets.

Alternative forage sources may also be needed for cattle during summer heat to compensate for the reduced feed intake resulting in decreased growth rate. Gutierrez, Alvarez, Arrizon, Carrasco, Salinas-Chavira and Zinn [18] found that providing cattle with an algae supplement (consisting predominantly of over 30 species of green and blue-green algae) increased average daily weight gain by 7.8% (*p* = 0.02), gain efficiency by 5.7% (*p* = 0.08) and estimated dietary net energy intake by 3.7% (*p* = 0.09) (Table 2). This is due to the supplement increasing the energy utilisation (*p* = 0.08), although the definitive cause is unknown and requires further analysis.

Costa, Quigley, Isherwood, McLennan and Poppi [68] also recorded linear increases in average daily weight gain in the cattle supplemented with Spirulina platensis (Table 2). Comparatively, the control cattle being fed a diet of spear-grass hay experienced weight loss, which was expected due to the low quality of this forage at 33 g crude protein per kilogram dry matter. This demonstrates that algae supplementation can improve forage quality by increasing the crude protein value to 675 g/kg dry matter. Further research is needed to fully understand the mechanistic action for these gains; however, these studies highlight that algae supplementation has the potential to be used as an additional supplement with low quality roughage to achieve positive weight gain results.

### 6.3. Antioxidant and Immune Modulating Effects

With mortality and morbidity being a worldwide issue for the beef industry there is high demand for supplements with immune enhancing antioxidant properties. Antioxidants possess the ability to scavenge free radicals. Free radicals are molecules that contain an unpaired electron capable of independent existence. Reactive oxygen species (ROS) are free radicals that are naturally produced biproducts from cellular respiration. Excess productions of ROS can damage important tissues and organs [16].

The blue-green algae species S. platensis was reported by Ghattas, Dawoud, Mahrous and Elgabry [19] as having immune modulating properties (Table 2). It was found that S. platensis supplementation in cattle increased plasma globulin levels (*p* < 0.05), protein and haemoglobin concentrations (*p* < 0.05), increased erythrocyte and leukocyte counts (*p* < 0.05). The increase in leucocytes is of great significance as they are involved in the innate or non-specific immune system which could be of benefit in reducing disease susceptibility and potentially BRD; however, further investigation is required to determine the efficacy of algae with BRD. Increases of these parameters were reported to be a result of enhanced haematopoiesis and improved absorption of the algae supplement due to its individual content of folic acid and vitamin B12. S. platensis supplementation also increased total antioxidant capacity (TAC) (TAC; *p* < 0.05) after 30 and 45 days, which was attributed to the presence of polysaccharides. This suggests that algae supplementation contains antioxidant and immunomodulatory properties. Furthermore, this immunomodulatory effect positively correlates with disease incidence. It was reported that there was a lower incidence of disease in the S. platensis supplemented cattle (6.67%) compared to the non-supplemented control group (9.72%) [19]. With algae containing bioactive compounds with antioxidant and immune modulating properties, it is speculated that they are contributing factors to the physiological effects seen by Ghattas, Dawoud, Mahrous and Elgabry [19]. However, further studies are required to evaluate these effects specifically from algae supplementation.

Flaga, Korytkowski, Gorka and Kowalski [10] reported that algal supplementation has potential anti-inflammatory effects with supplemented cattle recording lower levels of pro-inflammatory cytokines IL-1β, IL-6, and TNFα (Table 2). With a linear reduction in these cytokines and also in immunoglobin serum concentration IgG there is an observed correlation between increased algae supplementation and a decrease in inflammatory mediator levels. This study suggests that algae supplementation may be suitable to reducing the pro-inflammatory cytokines that contribute to pulmonary tissue damaged observed in cattle with BRD. Unfortunately, there are limited studies that investigate the effects algae supplementation have on pro-inflammatory cytokines within cattle. The anti-inflammatory effects of algae supplementation warrant further investigation to fully understand if algae supplementation is suitable as a complimentary nutraceutical preventative measure for BRD.

## 7. Palatability of Algae Supplementation in Beef Cattle

Algae has a strong aroma and taste which can decrease feed intake, ultimately decreasing daily weight gain. Flaga, Korytkowski, Gorka and Kowalski [10] investigated the effects of supplementing DHA rich algae (DHA-RA) Schizochytrium spp. in milk replacer in calves 8.6 ± 0.8 days old (Table 2). The study reported a decrease in daily intake of milk replacer mixed with the algae within the first few weeks of supplementation. This decrease was suspected to be due to the specific taste and/or smell of the supplement. Consequently, there was a 19.2% reduction in average daily weight gain of the supplemented calves compared to the control group. Overtime the animals became desensitised to the taste and smell of the algae supplement with the animals showing indifference to the supplement later in the study.

A reduction in palatability was also reported in a study by Drewery, et al. [73] (Table 2). Angus steers were fed a post-extraction algal residue (PEAR) in different carriers such as dried distillers’ grain (DDG), cottonseed meal (CSM) and liquid supplement (LS). The results showed that PEAR has a low palatability when fed with no carrier; however, when blended at levels up to 60%, there were no changes in feed intake indicating it was palatable to the animal. It is hypothesised by Drewery, Sawyer and Wickersham [73] that this decrease in palatability could be a result of the high sodium chloride content of the PEAR, with the contents measuring 52 g/kg Na, as rumen osmolality is directly related to feed intake. The presence of an effective feedback mechanism to reduce or slow intake of the supplement is also hypothesised in relation to regulation of the animal’s rumen osmolality. The supplement was provided at a level much lower than what would be expected for rumen osmolality to regulate feed intake, with the feedback mechanism being activated if osmotic pressure exceeds 400 mOsm/kg, indicating the additional consumption of commercial mineral blocks may be responsible for the short period of decreased feed intake. Although algae supplementation in cattle can cause a short period of reduced feed intake studies suggest upon adaptation to the smell and taste, feed intake and weight gain will resume.

## 8. Animal Product Quality

Due to increasing Australian consumer demand for high quality beef products there is considerable pressure on the industry to provide beef whilst also remaining cost effective [1]. Meat quality was assessed by Rossi, Compiani, Baldi, Taylor, Righi, Simoni and Quarantelli [69] through supplementing cattle with calcareous marine algae (CMA). The results showed that with the CMA supplementation the meat quality of the animal was significantly improved due to a reduced muscle pH, which as a result improves meat colour and tenderness which was tested using a shear force evaluation method (Table 2). The meat quality improvement was considered an indirect improvement of algae supplementation. The study observed a reduction in aggressive behaviour exhibited in the chute and pens which may have contributed to overall improved meat quality. The improvement in behaviour due to supplementation is not fully understood; however, it is suspected to be a result of the rumen buffering effect which ensures the rumen pH is at the optimum level between 6–7 [74]. This effect is shown by the desired increase in rumen pH to 6.25 (*p* < 0.01) compared to the sodium bicarbonate supplemented rumen pH of 5.62 [69]. As a result the supplemented cattle experienced lower incidence of a rumen pH level below 5.2 which is the level where ruminal acidosis is said to occur [74].

When an animal is placed under stress there is a release of catecholamines (epinephrine and nor-epinephrine) decreasing the overall meat quality. Catecholamine release increases the muscle pH, affects meat colour, firmness and also decreases the meats tenderness [69,75]. As a result, algae supplementation has the potential to be used within the beef industry to improve meat product quality.

## 9. Conclusions and Future Directions

Algae supplementation, specifically with *N. oculata*, provides various health benefits due to its bioactive compounds. These health benefits have the potential to reduce the incidence of diseases such as BRD by increasing antioxidant and reducing proinflammatory factors. A reduction in BRD incidence from both a financial and welfare prospective, would be beneficial to the Australian beef industry. With algae being economical to produce, it would serve as a feasible option as a feed supplement. However, future work in the complete analysis of the bioavailability of the bioactive compounds of *N. oculata* is still required. In addition, investigation of the in-vivo and in-vitro effects of algae supplementation on the biochemical and immunological parameters in beef cattle will provide further insight into the mechanisms of algae supplementation.

## Figures and Tables

**Table 1:** Major bacterial and viral species associated with BRD.

Bacteria Species	Viral Species	Reference
*Histophilus somni*	Bovine herpesvirus (BHV-1)	[25]
*Mannheimia haemolytica*	Bovine parainfluenza virus 3 (PIV)	[25]
*Pasteurella multocida*	Bovine respiratory syncytial virus (BRSV)	[25]
*Mycoplasma bovis*	Bovine viral diarrhoea virus (BVDV)	[25]
*Arcanobacterium pyogenes*		[30]

**Table 2:** Overview of the effects produced by different algae species in cattle.

Algae Species	Effect	Dose Rate Used of Algae	Study Design	Reference
*Schizochytrium* spp.	↓ feed intake ↓ average daily weight gain ↓ pro-inflammatory cytokine expression	9, 18, and 27 g DHA-RA/day	40 female Holstein-Friesian calves, divided into four groups, fed twice daily for 49 days.	[10]
*Chlorella* sp.	↓ feed intake ↑ N absorption and retention	1000 g/day	12 steers fed supplement for 4 days periods with a 3 days washout period.	[73]
*Spirulina platensis*	↑ leukocyte count ↑ plasma globulin concentration	6 g/day	16 Holstein calves, divided into a control and supplement group, fed for 45 days.	[19]
Green and blue-green algal biomass	↑ average daily weight gain ↑ weight gain efficiency	60 g/day	60 Holstein steers, divided into four experimental groups, 90 days trial.	[18]
*Lithothamnium calcareum*	↑ growth rate ↑ feed conversion ↓ acidosis incidence ↑ calmer behaviour traits	18.2 g/kg of DM and 15.5 g/kg of DM	180 Charolais bullocks, divided into two groups, 130 days trial.	[69]
Algae product containing 10% DHA	↑ pregnancy on first AI ↓ gestation period ↑ milk yield	100 g/day	1800 Holstein cows, primiparous and multiparous, 7-month trial.	[71]
*Spirulina platensis*	↑ average daily weight gain ↑ dry matter intake ↑ forage quality	4 g/kg	42 steers fed supplement with varying basal diets, 10 week trial with an additional 7 day adaptation period.	[68]

Note: ↑ = increase, ↓ = decrease.
